# Vitamin E deficiency and risk of equine motor neuron disease

**DOI:** 10.1186/1751-0147-49-17

**Published:** 2007-07-02

**Authors:** Hussni O Mohammed, Thomas J Divers, Brian A Summers, Alexander de Lahunta

**Affiliations:** 1 Department of Population Medicine and Diagnostic Science, College of Veterinary Medicine, Cornell University, Ithaca, NY 14853, USA; 2 Department of Clinical Sciences, College of Veterinary Medicine, Cornell University, Ithaca, NY 14853, USA; 3 Department of Molecular Medicine, College of Veterinary Medicine, Cornell University, Ithaca, NY 14853-6401, USA; 4 Currently, Royal Veterinary College, University of London, Fatfield, Herts AL9 7TA, UK

## Abstract

**Background:**

Equine motor neuron disease (EMND) is a spontaneous neurologic disorder of adult horses which results from the degeneration of motor neurons in the spinal cord and brain stem. Clinical manifestations, pathological findings, and epidemiologic attributes resemble those of human motor neuron disease (MND). As in MND the etiology of the disease is not known. We evaluated the predisposition role of vitamin E deficiency on the risk of EMND.

**Methods:**

Eleven horses at risk of EMND were identified and enrolled in a field trial at different times. The horses were maintained on a diet deficient in vitamin E and monitored periodically for levels of antioxidants – α-tocopherols, vitamins A, C, β-carotene, glutathione peroxidase (GSH-Px), and erythrocytic superoxide dismutase (SOD1). In addition to the self-control another parallel control group was included. Survival analysis was used to assess the probability of developing EMND past a specific period of time.

**Results:**

There was large variability in the levels of vitamins A and C, β-carotene, GSH-Px, and SOD1. Plasma vitamin E levels dropped significantly over time. Ten horses developed EMND within 44 months of enrollment. The median time to develop EMND was 38.5 months. None of the controls developed EMND.

**Conclusion:**

The study elucidated the role of vitamin E deficiency on the risk of EMND. Reproducing this disease in a natural animal model for the first time will enable us to carry out studies to test specific hypotheses regarding the mechanism by which the disease occurs.

## Background

Spontaneous motor neuron diseases are uncommon in domestic animals. Where they have been subject to study, these disorders invariably demonstrate a familial pattern, occurring in specific breeds of animals such as Brittany Spaniel dogs [1], Brown Swiss cattle [2] and Yorkshire pigs [3]. Clinical deficits are evident in the first year of life and often by a few months of age. The neuropathologic findings are a common theme of neurofilament accumulation in neurons and proximal axons, progressive motor neuron degeneration and spinal muscular atrophy. Accordingly, in 1990, considerable excitement accompanied the identification [4] of equine motor neuron disease (EMND), a sporadically occurring motor neuron disease affecting several horse breeds including standardbred, thoroughbred, Quarter horse and Arab breeds. The disorder presents in adult horses with a median age of 10 years. While EMND has been observed most frequently in the Quarter Horse breed, we believe that this is due to the manner in which these horses are housed and fed rather than by a primary genetic determination.

Equine motor neuron disease is a neurodegenerative disorder of the horse characterized by progressive weakness, fasciculations, muscle wasting, and weight loss [4,5]. Postmortem studies on afflicted horses revealed that weakness and muscle wasting result from degeneration of motor neurons in the spinal cord and brain stem [4]. The nature and the distribution of the neurodegenerative changes in EMND are strikingly similar to those reported in human progressive muscular atrophy, a form of amyotrophic lateral sclerosis (ALS) or Lou Gehrig's disease [6-8]. As in ALS, horses afflicted with EMND lose 30% of the somatic motor neurons in the spinal cord and the brain stem before they manifest clinical signs [9].

In the United States, EMND has been observed and reported widely but appears to be more common in the northeastern states [4,10]. The pattern of the disease is sporadic and typically in a group of horses on a farm, only a single animal is affected. The annual incidence of EMND in the U.S. varied by region and ranged from 0 in several regions to 2.78 per 100,000 horses in New England [10]. Worldwide, the disease has been recognized and documented in Canada, South America, Europe, and Asia [11-13]; exceptionally in one stable in Brazil, a high incidence has been noted.

Epidemiologic observational studies to date on EMND have established an association between the age of the horse and the risk of this disease [10,11,14]. The pattern of age association is similar to the one reported in the human MND [15] where older hosts are more susceptible to the disease.

Significant association was observed between a diet poor in vitamin E and the risk of EMND [5,14,16]. These field studies were corroborated with clinical laboratory and histopathological findings. Horses afflicted with EMND had significantly lower plasma vitamin E levels than normal horses either from the general population or stablemates [5,14]. Other evidence of hypovitaminosis E was found on direct and indirect ophthalmoscopic examination; affected horses reveal a pigmentary retinopathy which involves the retinal pigment epithelium. While the vision of affected horses appears normal, there are changes in the electroretinogram [13,17]. Furthermore, electron microscopic studies on the spinal cords of EMND animals have consistently demonstrated the presence of large endothelial accumulations of lipopigment granules [18]. In other species, endothelial accumulations of this nature have been identified as ceroids associated with vitamin E deficiency [20].

Despite the observations made, it is difficult to conclude that the observed association between vitamin E deficiency and the risk of EMND is causal because the samples in which determinations were made were collected at the same time the cases were diagnosed. We asked whether feeding horses a diet deficient in vitamin E would put them at risk for developing EMND. In other words, we evaluated the causal relationship between exposure to a diet that is low in vitamin E and the risk of EMND.

## Methods

### Study Design

We carried out a self-control (ie, each horse was its own control and the response to the intervention was compared to the baseline data) field trial to address the above-stated objectives. In this trial, normal horses potentially at risk of EMND were recruited, baseline data were collected, and the animals were followed for a period of time to acclimate the horses to the experimental environment before the studies began. The protocol for the undertaken studies was approved by the Institutional Animal Care and Use Committee at Cornell (Protocol # 94–23). All procedures have been in compliance with the institutional guidelines developed and monitored by the Center for Research and Animal Resources at Cornell.

#### Recruitment of Horses

The potential pool of horses to be considered for the trial originated from stables in the northeastern United States. Candidate horses were clinically examined and judged to be sound, especially with regards to neurologic function. Blood samples were drawn from candidate horses for determinations of levels of muscle enzymes and vitamin E.

In addition to the self-control design, another external control group was identified from horses that are kept at the Equine Research Park for teaching purposes. This group consisted of five horses which were randomly selected from a horse herd of 40 animals. This parallel control group was intended to control for the potential extraneous effect of the likelihood of EMND.

#### Inclusion Criteria

Candidate horses with low normal levels of vitamin E (<2.0 μg/ml (Table 1), normal muscle enzymes, and that were clinically sound were noted and considered at-risk. Plasma vitamin E levels were determined at least three consecutive times in the candidate horses over a month period. Horses that had persistently low-normal vitamin E values and were judged to be clinically normal were enrolled in the study. As clinical EMND cases typically (mode values) have plasma vitamin E levels < 0.5 μg/ml, the levels in candidate horses were twice higher. Only horses that met all the inclusion criteria were enrolled in the study. The rationale for targeting horses with low plasma vitamin E levels was to identify horses at risk and shortening the follow up period.

**Table 1:** Distribution of breed, weight, age, sex, plasma vitamin E levels of horses enrolled in the study

**Horse Identification**	**Breed**	**Weight (Kg)**	**Age (years)**	**Sex**	**Initial vitamin E values(μg/ml)**	**Follow-up period (month)**
801	Mixed	484	≥10^a^	Mare	1.3^b^	42
817	Quarterhorse	432	≥10	Mare	0.91	35
821	Mixed	381	≥10	Mare	1.4	33
833	Mixed	398	≥10	Mare	0.84	5*
980	Quarterhorse	522	13	Gelding	1.1	42
985	Thoroughbred	507	9	Mare	1.81	41
986	Mixed	396	≥10	Gelding	0.84	18
987	Thoroughbred	444	≥10	Gelding	1.38	41
988	Mixed	425	≥10	Mare	1.6	4
990	Mixed	392	16	Mare	1.44	29
991	Thoroughbred	450	10	Gelding	1.51	37

^a^Dental estimate

^b^Mean of four consecutive samples

#### Baseline data

Selected horses were transported to Cornell University's Equine Research Park, where they underwent complete physical examinations as well as initial plasma assays of antioxidants (vitamins A, C, and E, and β-carotene). Each horse received a clinical score according to a multidimensional scale that has several categories, including weight, evidence of muscle atrophy, weakness, short strides, trembling, recumbency, feet under body, sweating, head hanging, muscle fasciculation, shifting, tail slack, and collapse. All horses scored within the normal range. Also, all horses received a thorough eye exam and were scored accordingly. Biopsies from the dorsomedial sacrocaudalis muscle from five horses were examined to confirm their clinical status of being free of EMND [13]. The biopsies revealed no evidence of denervation atrophy.

### Laboratory procedures

#### Determination of β-carotene, α-tocopherol, and retinol levels in plasma

Aliquots (1 ml) of plasma were transferred to sterile, polypropylene, screw-cap microtubes with neoprene O rings (Sarstedt, Inc.) containing an antioxidant mixture (100 ml of an ethanolic mixture of propylgallate and EDTA) and held at -75°C until testing. The analyses were performed based on high-performance, liquid-liquid partition chromatography (HPLC). The analytes of interest were detected by spectrophotometery (450 nm for β-carotene for 1.38 min, 325 nm for retinol for 2.9 min, and molecular fluorescence emission at 330 nm for 7.05 min./α-tocopherol) using a tandem arrangement of two detectors, i.e., a variable-wavelength UV-Vis detector and a spectrofluorometric detector.

#### Determinations of Vitamin C concentration

All vitamin C plasma levels determination was performed at the Animal Health Diagnostic Laboratory (AHLD) at Cornell University using the HPLC analytical method for ASA described by Burtis and Ashwood [21]. An aliquot of 20 μml was injected into the HPLC. The HPLC system consisted of a spectrophotometric detector and a reverse phase HPLC column. The mobile phase was 1 mmol/L ammonium formate, 7 mmol/L dodecyltrimethylammonium bromide and 40% methanol (taken to pH 5.2 with formic acid). Elution was isocratic at a flow rate of 0.9 μL/min and the eluent was monitored at 265 nm.

#### Determination of glutathione peroxidase (GSHPx)

The activities and concentrations of GSHPx were determined using a modification of the method described by Paglia and Valentine [22]. The activities of GSHPx were measured as the production of NADP+ by the action of glutathione reductase (GR) on oxidized glutathione (GSSG) in the presence of NADPH.

#### Determination of superoxide dismutase (SOD1) in erythrocytes

The erythrocytic levels of superoxide dismutase (Cu, Zn-SOD1) were determined using the method described by Paoletti and Mocali [24]. Briefly, heparinized blood samples collected from horses were centrifuged to harvest the erythrocytes which were stored at -80°C until used. For assay, 500 μl of supernatant was treated with 800 μl of ethanol/chloroform extraction reagent (500 μl ethanol/300 μl chloroform). The mixture was vortexed for 30 seconds and then spun at 8500 g, resulting in two layers. The top, aqueous layer was further processed for the assay or frozen at -80°C until assay. Spectrophotometric assay of the SOD1 activity was based on the enzyme's ability to inhibit superoxide-driven NADH oxidation.

#### Maintenance of horses

At Cornell's Equine Research Park, the selected horses (n = 11) were maintained in an open stall (12 × 18 m) with a dirt floor and were given access to a wood-fenced dirt paddock (around two hectares). The horses had no access to pasture or green grass. All horses were fed a concentrate feed that was prepared according to National Research Council (NRC) guidelines, except vitamin E was not added. Chemical analysis was performed on this feed to determine the concentrations of vitamin E. The result showed that the feed contained < 11.98 mg/kg, which is an insignificant amount of dietary supplement (normal horse feed would contain 80 μg/kg vitamin E). Each horse received 2.5 quarts (about 5 lb, or 2.67 Kg) of this commercial feed a day. These horses also were fed mature-grass hay demonstrated to contain <10 mg/kg of vitamin E. The hay was provided *ad-lib*. All horses were followed for 44 months, and data on their antioxidant levels were routinely determined.

The external-control horses were managed similarly except that they had access to pasture and their concentrate feed included vitamin E. The hay was provided *ad-lib*. All control horses were judged to be clinically sound and vitamin E determinations were made in all of them. In addition vitamin A, C, β-carotenes, GSHPx, and SOD1 were also performed on the control horses.

#### Data collection protocol

Horses enrolled in the study were examined daily by the animal attendant for any abnormal clinical sign. The veterinarian was notified immediately if any of the horses manifested a clinical abnormality. Blood samples were collected at six-month intervals for determination of the antioxidant levels. Horses that developed clinical signs compatible with EMND also had blood samples collected and antioxidant levels determined. Horses succumbing to EMND or euthanized on the basis of humane considerations had a necropsy performed and the clinical diagnosis of EMND was confirmed by histopathological examination of the central nervous tissues for evidence of degenerations, such as glial scarring in the ventral gray column and Wallerian degeneration of the intramedullary portion of the somatic efferent neurons [4,13].

### Data analysis

The significance of changes in vitamins E, A, C, β-carotene, and GSHPx levels between baseline and end of study levels on the same horse were evaluated using the pair t-test. The changes in the activities of the SOD1 between baseline and end of the study were also evaluated using the pair t-test. Regression-analysis was used to determine the significance of change of the levels of the antioxidants in each horse. Rate of change was measured by the value of the respective regression coefficient. Comparisons between treatment and control groups were made using the t-test. All statistical hypotheses were tested at α = 0.05 (type I error).

Survival analysis technique was used to describe the distribution of EMND experience for the horses enrolled in the study. The distribution was summarized in terms of the survivor function, (the probability that a horse enrolled in the study would not develop EMND beyond a specified time period) and computed using the Kaplan and Meier method [24].

## Results

### Baseline data

**Figure 1:**
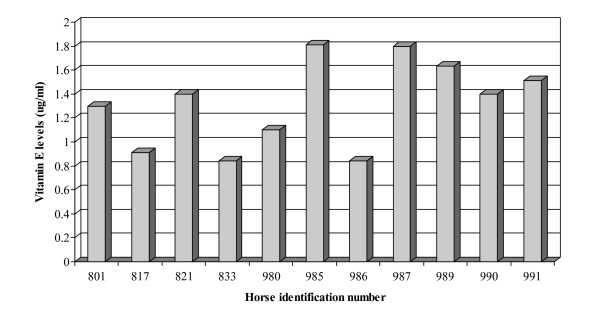
Initial plasma vitamin E levels. The mean value of 4 replicates is shown for each horse enrolled in the study.

Eleven horses met the inclusion criteria and were enrolled in the study. In the recruitment process, we screened 30 horses for vitamin E plasma levels before deciding on the eleven enrolled. All eleven horses had normal clinical scores at enrollment. The distribution of breed, age, sex, and weight of the enrolled horses is shown in Table 1. The initial plasma vitamin E levels ranged from 0.84 to 1.81 μg/ml with a median value of 1.4 μg/ml (Figure 1). There was no significant difference in vitamin E levels among treatment (self-control) horses. There was no significant difference in vitamin E levels among (parallel) controls (median = 2.81; range 1.44 – 3.06 μg/ml).

**Figure 2:**
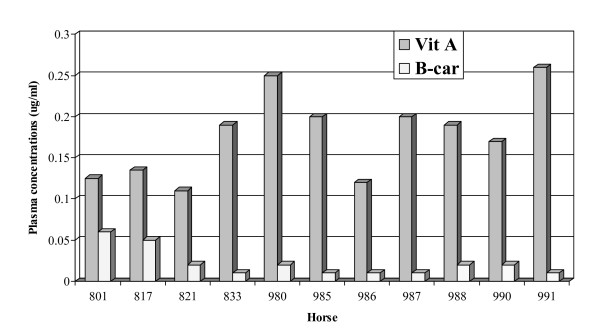
Plasma levels of vitamin A and β-carotene. Mean values of four replicates are shown for each horse at the time of enrollment.

Initial plasma vitamin A levels ranged from 0.10 to 0.26 μg/ml (median = 0.15 μg/ml (Figure 2). Plasma β-carotene levels were similar among the horses in the two groups (parallel and self-control) (median = 0.01, range = 0.005, 0.06 μg/ml). The median vitamin C level was 2.27 μg/ml (range 1.7 to 3.2 μg/ml). There was no significant variation in the blood GSH-Px values among horses (1.594 ± 0.33 μg/ml). All horses in the treatment group had similar SOD1 activities (1.91 units ± 0.68).

Initial plasma vitamin A levels for the control horses ranged from 0.18 to 0.3 μg/ml (median = 0.185 μg/ml). Plasma β-carotene levels were similar among the control horses (median = 0.043, range = 0.02, 1.14 μg/ml). The median vitamin C level for the control horses was 2.33 μg/ml. There was no significant variation in the blood GSH-Px values among the control horses (1.454 ± 0.32 μg/ml). The control horses had similar SOD1 activities (1.96 units ± 0.63).

### Follow-up

One horse was lost from the study after being enrolled for five months; the horse (# 833) died because of septicemia resulting from bacterial infection in the elbow. Histopathological examinations of the nervous tissues showed no evidence of EMND in this horse. This horse was replaced with another that met the aforementioned inclusion criteria (# 991) (Table 1).

**Figure 3:**
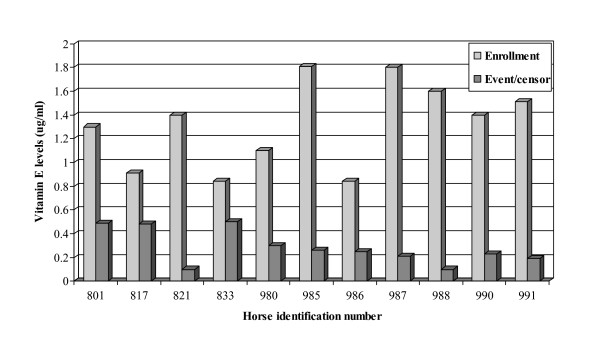
Changes of plasma vitamin E levels. Mean value of vitamin E at initial enrolment and at censoring for each horse in the study. The significance of changes in the mean values was evaluated using paired *t*-test. The mean of the changes was significantly different from zero.

**Figure 4:**
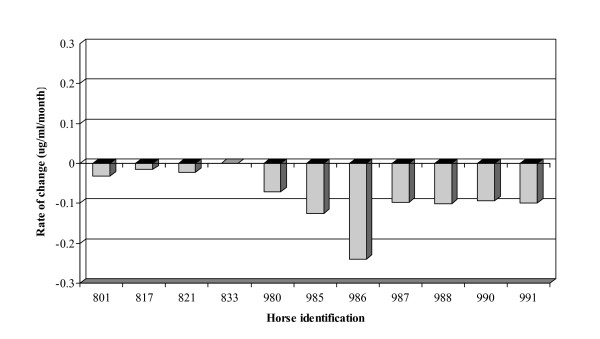
Rate of change in plasma vitamin E levels. The significance of the coefficient was determined using the *t*-test. All of the coefficients were significantly different from zero except for horse number 833.

Plasma vitamin E levels dropped significantly in all horses enrolled in the deficient (self-control) group as evaluated by the paired t-test (Figure 3). The median percent change from baseline to end of enrollment in plasma vitamin E levels was 82 % (range = 47 – 93 %). Figure 4 shows the rate of change in vitamin E levels (μg/ml) as estimated from the regression analysis. The average rate of change was -0.14 (95 % CI (confidence interval) -0.02, -0.26).

**Figure 5:**
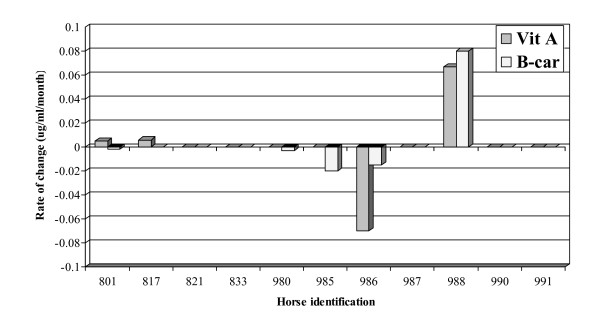
Rate of change (regression coefficient) in vitamin A and β-carotene (B-car). The significance of the coefficient was determined using the *t*-test. None of the rate of changes was significantly different from zero. (Regression coefficient reflects the changes in plasma vitamin A and β-carotene per day).

There were no significant changes in levels of vitamin A, C (data not shown), or β-carotene for all horses in the deficient group (Figure 5). There was a variation in the levels of GSH-Px in horses enrolled in the study over time, but the changes were not significant. There were no significant changes in SOD1 activities in the horses between enrollment and end of the study.

### Risk of EMND

Overt clinical signs of EMND were observed in 3 horses. The first horse that showed clinical signs consistent with EMND was at 18 months post-enrollment. The affected horse demonstrated the typical clinical signs of progressive weakness, muscle fasciculations, tremor, and wasting. This horse was euthanized, and the diagnosis of EMND was confirmed by histopathologic examination of the spinal cord and brain stem. A second and third horse were diagnosed with EMND after 29 and 33 months of enrollment, respectively. The pathological changes were found most consistently and abundantly in the ventral horns of the spinal cord and certain motor nuclei of the cranial nerves. Degeneration was less abundant in the spinal ganglia.

One of the horses enrolled in the study experienced severe colic one night, 35 months post-enrollment, and had to be euthanized. This horse did not show any clinical sign suspecting of EMND before the episode of colic; however, nerve tissues collected showed pathological changes that were consistent with EMND. A fifth horse showed muscle fasiculations thirty-seven months in the study and a nerve biopsy was taken for confirmation. The biopsy was not conclusive and the horse was euthanatized one month later. The diagnosis of EMND was confirmed by examining the CNS.

**Figure 6:**
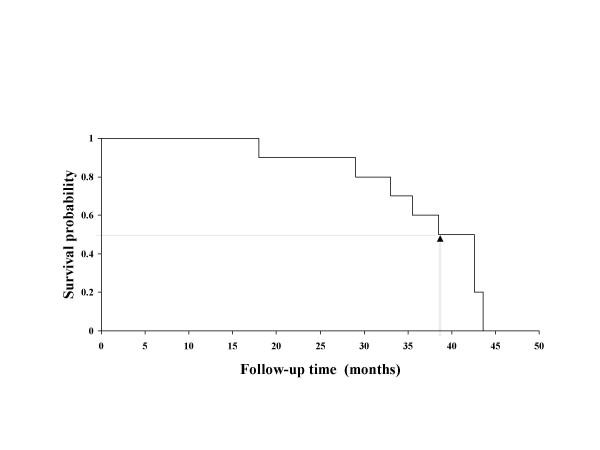
Survival rates. Plot of the survival experience of horses computed using the Kaplan-Meier method.

At the conclusion of the follow up period, none of the remaining five horses showed overt clinical signs suspecting of EMND, and each had a normal clinical score. Spinal-accessory-nerve biopsies were collected from these horses 41–42 months post-enrollment and examined histopathologically for evidence of EMND [13]. All horses showed pathological changes that were consistent with the diagnosis of EMND. Figure 6 shows the survival experience of all horses enrolled in the study. The median time to develop EMND was 38.5 months (95 percent confidence interval for the median was 33.5, 42.6 months).

None of the external controls that were maintained at the Equine Research Park developed EMND. Vitamin A, β-carotene, and vitamin C value did not vary significantly between initial enrollment and right censoring (end of the study) or the control horses. The median and range values at the end the study were 0.151 μg/ml (range = 0.175 – 0.30), 0.054 μg/ml (range = 0.028 – 0.08), and 3.28 μg/ml (range = 2.27 – 4.54).

## Discussion

In the years after 1990, the newly identified EMND was viewed as sharing clinical and neuropathologic features with human MND [4,11] While on epidemiologic grounds, the equine disease appeared to be purely sporadic, we decided to examine equine SOD1 for polymorphisms given the association of mutations in this gene and familial human MND. No association between SOD1 variants and EMND were found [25]. In contrast, our field visits to farms with EMND cases suggested a connection between certain dietary practices and the disorder. We found cases of EMND commonly where horses had no access to pasture or other green feed and were fed poor quality food/hay [5,10,14]. Specifically, we performed this study to investigate a possible causal relation between a dietary deficiency of vitamin E and the risk of EMND. The previous evidence was built through observational studies and, by virtue of their nature, it is impossible to establish causal relationship between the deficiency in this antioxidant and the risk of EMND [26]. Vitamin E determinations on the EMND-afflicted cases and control horses in the prior observational studies were made at the time of disease diagnosis. At such a time in the course of this motor neuron disease, it is impossible to discern which took place first, the deficiency in the antioxidant or the development of the disease. Therefore, it was important to carry out this dietary trial to establish the chronological sequence of events and confirm the suspected causal relationship between the deficiency and the risk of the disease.

We adopted a field trial design in which we used the horse as its own control to assess the impact of the vitamin E deficiency on the same animals and hence minimize the potential effect of other intrinsic factors. In addition an external control group was identified to control for the effect of extraneous factors that might predispose horses to the risk of EMND.

In most mammalian species including the horse, uncomplicated vitamin E deficiency is almost exclusively an axonal degenerative disease in the young or a pigmentary retinal disorder. The axonopathic effect is characterized by dystrophic changes in the distal axons of spinal proprioceptive tracts with little or more commonly no involvement of motor neurons [19,27]. Our work and that of others on equine degenerative myeloencephalopathay (EDM) [19], a disease of young horses (1 to 3 years), fails to document similar distal axon changes in adult horses severely deficient of vitamin E. Furthermore, all the horses enrolled in this study exceeded the age at which they would be at greatest risk for EDM (horses typically under 3 years of age). All of the horses enrolled in the study developed the characteristic retinal degeneration that associated with prolonged vitamin E deficiency [19].

Vitamin E is essential for the integrity and optimum function of several systems in the body, including nervous, immune, reproductive, muscular and circulatory systems [28]. Several and varied human diseases have been attributed to deficiency in vitamin E, including ischemic heart disease, atherosclerosis, diabetes, cataract, Parkinson's disease, Alzheimer's disease, and other neurologic disorders including ALS [28-30]. Vitamin E is a known antioxidant that helps in the neutralization of free radicals [28]. This antioxidant activity is seen as the underlying factor in most of vitamin E functions – vitamin E blocks the chain reaction of lipid peroxidation by scavenging the intermediate peroxyl radical that is produced in the reaction [31]. In this trial all the horses had a significant reduction in vitamin E levels for a relatively long period of time (approximately 3+ years). The severe and chronic deficiency in vitamin E would put horses at risk of oxidative stress as a result of reduction in antioxidant capacity. We investigated the hypothesis of oxidative stress in a different study where the production of free radicals was exacerbated by feeding vitamin E deficient horses a diet that was supplemented with prooxidants, copper and iron [16]. Experimental horses developed EMND at a faster rate than in this current study. No supplements were added to the diet in the current study.

There is mounting evidence of a role for oxidative stress in the risk of human motor neuron disease [32,33]. Studies on cases of familial ALS (FALS) indicate a pathogenesis related to dominantly inherited point-mutations in the gene for Cu, Zn superoxide dismutase (SOD1) on chromosome 21 [34,35]. The nature of the toxic gain of function caused by the SOD1 mutation in FALS has been elusive [36,37], yet recent studies [34] find that the mutated gene in transgenic mice places the CNS under oxidative stress, which secondarily causes a deficiency of vitamin E [34]. No significant association between SOD1 and the risk of EMND was observed in the current study.

We have found significantly lower levels of plasma and nervous-tissue levels of vitamin E in EMND cases in comparison to controls (16). All horses enrolled in this trial also had a significant drop in plasma vitamin E levels. However, findings on vitamin E levels in the human motor neuron disease are not conclusive. This discrepancy could be attributed to several factors including the nutritional uptake of the patients at the time of diagnosis. The disease has a relatively long time between onset and diagnosis in humans. It is estimated that the average duration between onset and diagnosis of ALS is 12 month [38]. Because the clinical signs are typified by weakness and muscle loss, it is more likely that the patients would react to the symptoms by changing their dietary intake – which is likely to include supplementation of minerals and vitamins, including vitamin E. In spite of the discrepancy in reporting the plasma and CSF levels of vitamin E in SALS patients, there is a consensus that there is increased lipid peroxidation in the disease [30,40-42].

There has been a long-term interest in vitamin E because of its role in the integrity of membranes and its association with deficiency syndromes that included encephalomalacia and muscle weakness in man and animals [20,39,43]. Such findings have led to exploring its potential in the therapy of ALS. Although there was an excitement about its therapeutic effect in 1940s [43,44], the excitement was tempered by the failure of reproducing the findings in later studies [46].

The levels of other antioxidants in horses enrolled in this study – vitamins A and C, β-carotene, and GSH-Px – did not change significantly. This finding is consistent with reports on human ALS, where the studies found no significant differences in the plasma levels of vitamin A, β-carotenes, and glutathione peroxidase [30,47,48]. β-carotene, a precursor of vitamin A, is known to have an important antioxidant activity. Although there were no significant changes in vitamin A levels in the horses enrolled in the study, three horses developed retinal pigmentation. One of the horses had undetectable levels of plasma vitamin A.

In this study we found no significant difference between and within horses in relation to the activities of the SOD1 enzyme. These determinations were made over the course of the study period at predetermined intervals. The last determinations were made at the onset of the clinical signs or at censoring. Genetic studies on this disease failed to show polymorphism in the SOD1 gene [25]. In humans, decreased activities of the SOD1 enzyme were reported in FALS patients [39]. The SOD1 findings in this study are similar to the observation made on the human SALS.

A retrospective study by McGorum et al., [49] reported that horses on pasture in Scotland were at risk of EMND. The inference in their study was that access to pasture is a good indicator for availability of vitamin E. However, the authors indicated that many of the affected horses had low plasma vitamin E levels. Such a finding of low vitamin E plasma levels in those horses invite several speculative explanations including the quality of the pasture, bioavailability of vitamin E, and the health of the horse in terms of absorption capacity. In our study we carried out a controlled field trial to avoid speculative conclusions.

As a spontaneous, sporadic, and progressive degenerative disorder of bulbospinal motor neurons, EMND bears close resemblance to SALS [4]. Unlike other spontaneous animal models, EMND has many clinical, pathological, and epidemiological features of SALS. Nevertheless, as a spontaneous animal model EMND offers the prospect those epidemiologic studies possibly will identify risk factors with bearing on the pathogenesis of SALS. Moreover, and in light of this experimental finding where we are able to reproduce the disease, EMND offers the opportunity to test specific hypotheses and perform procedures that are not possible to do in humans.

## Conclusion

We believe that EMND, just as ALS, may have a multifactorial etiology and that oxidative stress is a major contributing/predisposing factor, i.e., sufficient cause, in motor neuron death but not necessarily the sole etiologic agent/factor. While the dietary practices which appear to favor the development of hypovitaminosis E in horses are not new, EMND was not identified prior to 1990. This would suggest that more than vitamin E deficiency is in play. By reproducing the disease, we are an in a position to test specific hypotheses regarding the etiologic factor(s) while taking into consideration the role of oxidative stress. Through these etiologic studies we will be able to understand the pathogeneses of the motor neuron disease and may be able to provide new therapeutic avenues either by amelioration of the etiologic agent(s) or enforcement of the oxidative defense.

We believe that the results of our study represent a breakthrough in the advancement of the knowledge on the etiology and pathogenesis of EMND. The success of our efforts in reproducing this disease in a natural model offers a unique opportunity with great implications to human health in general and ALS in particular. The findings in this study will allow us both to focus on testing specific etiologic hypotheses that will add to the understanding of this disease and to evaluate critical intervention(s) that can contribute to the treatment and prevention of the condition.

## Authors' contributions

HM conceived the study, developed the experimental design in collaboration with the authors, coordinated the different activities, performed the statistical analyses, and drafted the manuscript. TD participated in the development of the design, recruited the horses for the study, oversee the implementation of the field trial, and performed the clinical diagnosis; BS carried out the histopathological studies in collaboration with AD. AD performed the neurological diagnosis, carried out the postmortem studies and histopathological studies. All authors read and provide the final draft of the manuscript.
